# High-Intensity Interval Training in Older Adults With Treatment Naive Chronic Lymphocytic Leukemia

**DOI:** 10.1093/geroni/igab046.1769

**Published:** 2021-12-17

**Authors:** David Bartlett, Grace MacDonald, Mike Deal, Erik Hanson, Carl Pieper, J Brice Weinberg, Danielle Brander, Andrea Sitlinger

**Affiliations:** 1 Duke University, Durham, North Carolina, United States; 2 University of North Carolina, Chapel Hill, North Carolina, United States

## Abstract

Chronic lymphocytic leukemia (CLL) is the most common leukemia, affecting predominantly older adults. Treatment naïve patients (CLLtn) with low physical fitness have poor survival following commencement of treatment. CLLtn is characterized by inadequate immune functions, increased risk of secondary malignancies and infections. The aims of this study were to determine the feasibility and preliminary effects of 12-weeks of high-intensity interval training (HIIT) on CLLtn patients. We enrolled eighteen CLLtn patients (64.9±9.1yrs.). Eleven (5M/6F) were allocated to HIIT and seven (4M/3F) to the control group (CON). HIIT consisted of three 30-minute treadmill sessions/week plus two 30-minute strength training sessions/week. Feasibility was confirmed if >70% of HIIT participants completed >75% of prescribed sessions and prescribed minutes, and if >80% of high-intensity intervals were at a heart rate corresponding to 80% of aerobic capacity (139±19 bpm). Results are presented as mean±SD and effect sizes (d), with 0.2, 0.5 and 0.8 representing small, medium and large effect sizes, respectively. Feasibility was achieved, with HIIT completing 5.0±0.2 sessions/week and 99±3.6% of prescribed minutes/week at 142±19 bpm. No adverse safety events were observed. Compared to CON, HIIT increased leg (d=2.602), chest (d=1.285), and seated row (d=3.323) strength, while aerobic capacity difference between groups was d=0.431. Compared to CON, HIIT increased in vitro natural killer immune cell cytolytic activity against K562 (d=1.586) and OSU-CLL (d=0.917) cancer cell lines, and autologous CLL cells (d=1.362). HIIT is safe and feasible in older adults with CLLtn. Preliminary effects suggest that HIIT increases muscle strength and important components of immune function.

